# Feasibilty of Transcutaneous pCO_2_ Monitoring During Immediate Transition After Birth—A Prospective Observational Study

**DOI:** 10.3389/fped.2020.00011

**Published:** 2020-01-29

**Authors:** Ilia Bresesti, Marlies Bruckner, Christian Mattersberger, Nariae Baik-Schneditz, Bernhard Schwaberger, Lukas Mileder, Alexander Avian, Berndt Urlesberger, Gerhard Pichler

**Affiliations:** ^1^Research Unit for Neonatal Micro- and Macrocirculation, Department of Pediatrics, Medical University of Graz, Graz, Austria; ^2^Division of Neonatology, Department of Pediatrics, Medical University of Graz, Graz, Austria; ^3^NICU “V. Buzzi” Children's Hospital, ASST-FBF-Sacco, Milan, Italy; ^4^Institute for Medical Informatics, Statistics and Documentation, Medical University of Graz, Graz, Austria

**Keywords:** transcutaneous, carbon dioxide, neonate, transition, delivery room

## Abstract

**Background:** According to recommendations, non-invasive monitoring during neonatal resuscitation after birth includes heart rate (HR) and oxygen saturation (SpO_2_). Continuous transcutaneous monitoring of carbon dioxide partial pressure (tcpCO_2_) may further offer quantitative information on neonatal respiratory status.

**Objective:** We aimed to investigate feasibility of tcpCO_2_ measurements in the delivery room during immediate neonatal transition and to compare the course of tcpCO_2_ between stable term and preterm infants.

**Methods:** Neonates without need for cardio-respiratory intervention during immediate transition after birth were enrolled in a prospective observational study. In these term and preterm neonates, we measured HR and SpO_2_ by pulse oximetry on the right wrist and tcpCO_2_ with the sensor applied on the left hemithorax during the first 15 min after birth. Courses of tcpCO_2_ were analyzed in term and preterm neonates and groups were compared.

**Results:** Fifty-three term (gestational age: 38.8 ± 0.9 weeks) and 13 preterm neonates (gestational age: 34.1 ± 1.5 weeks) were included. First tcpCO_2_ values were achieved in both groups at minute 4 after birth, which reached a stable plateau after the equilibration phase at minute 9. Mean tcpCO_2_ values 15 min after birth were 46.2 (95% CI 34.5–57.8) mmHg in term neonates and 48.5 (95%CI 43.0–54.1) mmHg in preterm neonates. Preterm and term infants did not show significant differences in the tcpCO_2_ values at any time point.

**Conclusion:** This study demonstrates that tcpCO_2_ measurement is feasible during immediate neonatal transition after birth and that tcpCO_2_ values were comparable in stable term and preterm neonates.

## Introduction

Neonatologist are confronted with the complexity of monitoring transition from intra- to extra-uterine life. It is a multi-step event, characterized by rapid physiological changes in respiratory, cardio-circulatory and metabolic status.

The decrease in partial pressure of oxygen and the increase in that of carbon dioxide (pCO_2_) followed by respiratory acidosis is one of the main triggers for initiation of breathing (1). After proper and effective establishment of respiratory function, the exchange of CO_2_ and O_2_ should reach a steady state.

To date, there are different methods and devices to assess oxygenation and cardio-circulatory status during neonatal transition. The most widespread monitoring is pulse oximetry, a non-invasive mode to detect heart rate (HR) and peripheral oxygen saturation (SpO_2_). It is recommended as standard practice in the delivery room (DR) by current international guidelines (2). Reference values for both parameters during immediate transition are available (3, 4).

Although gas exchange problems may be frequent events during immediate post-natal transition, there is currently no evidence on the role of CO_2_ and continuous pCO_2_ monitoring in the DR. Moreover, uncertainty remains about its feasibility and reliability in this setting.

In this study we aimed to evaluate the feasibility of transcutaneous pCO_2_ (tcpCO_2_) monitoring in stable neonates during the first 15 min after birth. Moreover, we compared tcpCO_2_ values between term and preterm neonates without need for any intervention immediately after birth. We hypothesized that tcpCO_2_ monitoring during immediate transition after birth is feasible and that term and preterm neonates differ in tcpCO_2_ during this phase.

## Methods

A prospective observational study was conducted from October 2015 to June 2018 at the Division of Neonatology, Department of Pediatrics and Adolescent Medicine, Medical University of Graz, Austria. Institutional ethical approval (EC number: 27-465 ex 14/15) and written parental consent were obtained before inclusion. Term and preterm neonates born by cesarean section without any intervention immediately after birth were included in the study. Exclusion criteria for this analysis were congenital malformations and/or need for cardio-respiratory support.

Monitoring was performed during the first 15 min after birth. As soon as the neonate was placed on the resuscitation table in supine position, a pulse oximeter probe was applied on the right wrist and HR and SpO_2_ were measured using the IntelliVue MP50 monitor (Philips, The Netherlands). The tcpCO_2_ sensor (TC Sensor 84, Radiometer RSCH GmbH, Thalwil, Switzerland) was applied on the infant's left hemithorax and values were displayed on a separate monitor (IntelliVue MP70, Philips, The Netherlands). In accordance with the manufacturer's guidelines, the sensor's temperature was set at 39°C, the lowest level for tcpCO_2_ measurements. The fixation of the tcpCO_2_ sensor was observed continuously and data were only recorded when the sensor was in place and remained attached to the skin.

### Statistical Analysis

Demographic variables are presented as absolute counts, mean and standard deviation (SD) or median and interquartile range (IQR), as appropriate. Comparisons of categorical baseline characteristics between preterm and term neonates were made using chi-square test, *t*-test or Mann Whitney *U*-test, as appropriate. tcpCO_2_, SpO_2_, and HR data are presented as mean and 95% confidence interval (95% CI). We investigated tcpCO_2_, SpO_2_, and HR across time within the first 15 min using a linear mixed model with fixed effects for time and gestational age (preterm vs. term neonates). A first order autoregressive covariance structure was used. The autoregressive covariance structure assumes a systematically decreasing correlation with increasing distance between time points. Therefore, adjacent time points have the highest correlations. *Post-hoc* analyses for differences between groups at each minute were performed for the comparison of gestational age groups (pre-term vs. term neonates). A *p*-value < 0.05 was considered statistically significant. Statistical analysis was performed using SPSS Statistics 24.0.0 (IBM, USA).

## Results

Out of 197 measured neonates in the DR, this study included 53 term and 13 preterm neonates without any cardio-respiratory intervention immediately after birth (Table 1).

**Table 1:** Demographic data of term and preterm neonates with tcpCO_2_ monitoring immediately after birth.

	**Term infants (*n* = 53)**	**Preterm infants (*n* = 13)**
Birth weight (g)	3,027 ± 473	1,880 ± 270
Gestational age (weeks)	38.8 ± 0.9	34.1 ± 1.5
**Gender (*****n*****)**		
Male	29 (55%)	4 (31%)
Female	24 (45%)	9 (69%)
Apgar score at min 1	9 (9–9)	9 (9–9)
Apgar score at min 5	10 (10–10)	9 (10–10)
Apgar score at min 10	10 (10–10)	10 (10–10)
Umbilical artery pH	7.31 ± 0.04	7.29 ± 0.05

*Data are expressed as absolute counts (%) or mean ± SD or median (IQR)*.

**Figure 1:**
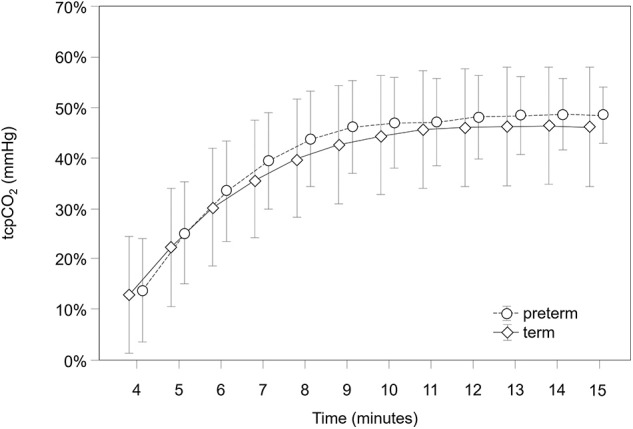
tcpCO_2_ course during the first 15 min after birth in term and preterm neonates.

Transcutaneous pCO_2_ values were analyzed from minute 4 after birth, which we considered the first proper detection time. Earlier values were detected in just a few patients; thus, they were not included in the analysis. Values reached a stable plateau after the equilibration phase at minute 9. Mean tcpCO_2_ values 15 min after birth were 46.2 (95% CI 34.5–57.8) mmHg in term neonates and 48.5 (43.0–54.1) mmHg in preterm infants. They both showed comparable courses of tcpCO_2_ without significant differences at any time point (*p* = 0.812) (Table 2 and Figure 1).

**Table 2:** tcpCO_2_ during the first 15 min after birth in term and preterm neonates.

**Minute**	**Term infants**	**Preterm infants**	***p*-Value**
4	12.8 (1.1–24.5)	13.8 (3.5–24.1)	0.752
5	22.4 (10.7–34.0)	25.1 (15.0–35.2)	0.386
6	30.3 (18.6–42.0)	33.5 (23.6–43.4)	0.312
7	35.7 (24.1–47.4)	39.5 (29.8–49.1)	0.229
8	39.9 (28.3–51.6)	43.8 (34.4–53.3)	0.208
9	42.7 (31.0–54.3)	46.2 (37.0–55.4)	0.262
10	44.4 (32.8–56.1)	47 (38.1–55.9)	0.409
11	45.7 (34.0–57.3)	47.2 (38.6–55.7)	0.630
12	46.1 (34.5–57.8)	48.1 (39.9–56.3)	0.531
13	46.3 (34.6–58.0)	48.5 (40.7–56.2)	0.487
14	46.4 (34.8–58.1)	48.7 (41.6–55.8)	0.467
15	46.2 (34.5–57.8)	48.5 (43.0–54.1)	0.442

*Mean values and 95%CI are presented*.

SpO_2_ and HR values were within targets (2), although SpO_2_ values in preterm neonates were significantly higher at minutes 2, 4, and 5 (in preterm infants at min 2: 75.8, 95% CI: 55.7–84.9, at min 4: 82.7, 95% CI: 73.9–91.5; at min 5: 87.3 95%CI: 78.6–96.0; in term infants at min 2: 68.7, 95% CI: 60.4–77.0, at min 4: 78.3, 95% CI: 70.0–86.5, at min 5: 87.3 95% CI: 82.5–90.8) and HR values were significantly lower at minute 2 (in preterm infants: 106 bpm, 95% CI: 71.5–141.9; in term infants: 138.3 bpm, 95% CI: 107.4–169.2).

## Discussion

To our knowledge, this is the first description of tcpCO_2_ measurements in term and preterm neonates in the DR during immediate post-natal transition (5). In this study, we demonstrated that the use of tcpCO_2_ monitoring is feasible and safe.

First values could be obtained rather quickly at minute 4 after birth. We observed a progressive increase of values over the first minutes, until a plateau was reached at minute 9 after birth. This initial trend of tcpCO_2_ is attributed to the equilibration phase of the skin sensor which is necessary to obtain reliable values.

As we only enrolled neonates who did not require cardio-respiratory intervention and completed post-natal transition in a physiological way, it is reasonable to assume that the observed tcpCO_2_ values reflect uncompromised neonatal transition.

The importance of pCO_2_ levels in neonates is well-known. In fact, evidence clearly shows that pCO_2_ strongly influences cerebral perfusion in newborn infants, especially in those of low gestational age (6), and its uncontrolled increase may lead to brain injury. Moreover, the fact that blood gas values change quite rapidly has raised interest for continuous monitoring of pCO_2_ (7). Therefore, invasive and non-invasive pCO_2_ monitoring is widely used in neonates admitted to neonatal intensive care units (NICU). Two non-invasive methods for pCO_2_ monitoring are available: the transcutaneous measurement and the exhaled or end-tidal CO_2_ (etCO_2_) measurement (8, 9). They both provide continuous estimation of pCO_2_ and they significantly reduce blood samples, which is particularly important in neonates. However, they also have some limitations mainly due to technical reasons.

EtCO_2_ monitoring during immediate transition has shown valuable results in neonates needing respiratory support (10) but its use is limited to ventilated patients. It reflects the efficacy of lung aeration and the establishment of effective V/Q match in the first minutes after birth and it seems to be a promising instrument in the DR more to confirm correct endotracheal tube placement or to verify the efficacy of mask ventilation (5). However, it is not that accurate in providing information on arterial pCO_2_ levels when neonates experience respiratory distress. This condition, peculiar of preterm infants, leads to poor pulmonary perfusion and compromises gas exchange. Therefore, the etCO_2_ device may underestimate high arterial pCO_2_ levels. Transcutaneous pCO_2_ might overcome this limitation and, if combined with etCO_2_, could contribute to a more reliable estimation of respiratory gas imbalances also in ventilated patients.

Of note, reference ranges for etCO_2_ in neonates during post-natal transition without resuscitation have been proposed recently (11).

Transcutaneous pCO_2_ monitoring is used in neonates during NICU care and relies on the fact that CO_2_ propagates through tissue and can be measured by a skin sensor. However, the correlation between tcpCO_2_ and blood pCO_2_ is related to the perfusion of the capillary bed, so heating of the skin sensor is required. However, this may cause skin burns. On the other side, the use of low sensor temperature has been correlated to an overestimation of the pCO_2_ (12).

In this study, no skin burns or other adverse reactions were observed, mainly due to the short measurement period in the DR, reducing the exposure of neonatal skin to high sensor temperatures.

This study also demonstrated that there was no difference between stable term and preterm neonates in regard to tcpCO_2_ courses and values during the first 15 min. The inclusion of neonates without need for cardio-respiratory support regardless of gestational age could in part explain this observation, even if these two populations are known to differ significantly in HR and SpO_2_ values during immediate transition, as also shown by our findings (3).

The present study has some limitations. First, we attributed the initial increase to the equilibration phase of the sensor. The current data set does not allow to differentiate exactly between equilibration of temperature, microcirculation and pCO_2_ of the tissue at the sensor site and true changes in arterial blood of the neonate. However, according the manufacturer's equilibration time and the observed increase of tcpCO_2_ to a stable plateau at minute 9 it can be assumed that thereafter tcpCO_2_ corresponds to pCO_2_ in the blood. Furthermore, even if this delay in acquisition of data cannot currently be avoided, gathering reliable data within 10 min after birth may still be helpful to guide medical DR interventions. Second, we did not compare tcpCO_2_ values and those measured from blood samples in the present analyses of neonates without any intervention. Since there is controversial evidence in this regard (13–15), this aspect warrants further investigation.

## Conclusion

Non-invasive measurement of tcpCO_2_ seems to be feasible during immediate neonatal transition, even if measurements in the very first minutes after birth remain challenging. Further research is desirable to confirm our results and to investigate any clinical impact of additional tcpCO_2_ measurements on DR interventions in neonates with adverse conditions during the transitional period.

## Data Availability Statement

The datasets analyzed in this article are not publicly available. Requests to access the datasets should be directed to Gerhard Pichler, gerhard.pichler@medunigraz.at.

## Ethics Statement

This study was carried out in accordance with the recommendations of Regional Committee on Biomedical Research Ethics with written informed consent from all subjects. All parents gave written informed consent in accordance with the Declaration of Helsinki. The protocol was approved by the Ethikkommission, Medizinische Universität Graz, Austria (EC number: 27-465 ex 14/15).

## Author Contributions

IB, GP, and BU: conception and design. IB, MB, CM, NB-S, BS, LM, and GP: collection and assembly of data. IB, MB, CM, NB-S, BS, LM, AA, BU, and GP: analyses and interpretation of data, critical revision, editing, and final approval of the article. IB, AA, and GP: drafting of the article.

### Conflict of Interest

The authors declare that the research was conducted in the absence of any commercial or financial relationships that could be construed as a potential conflict of interest.

## Abbreviations

HR: heart rate
NICU: neonatal intensive care unit
SpO_2_: arterial oxygen saturation
tcpCO_2_: transcutaneous monitoring of carbon dioxide partial pressure
DR: delivery room.
